# Complete Genome Sequences of Seven *Helicoverpa armigera* SNPV-AC53-Derived Strains

**DOI:** 10.1128/genomeA.00260-16

**Published:** 2016-05-05

**Authors:** Christopher Noune, Caroline Hauxwell

**Affiliations:** Queensland University of Technology, Brisbane, Australia

## Abstract

Wild-type baculovirus isolates typically consist of multiple strains. We report the full genome sequences of seven alphabaculovirus strains derived by passage through tissue culture from *Helicoverpa armigera* SNPV-AC53 (KJ909666).

## GENOME ANNOUNCEMENT

Wild-type baculovirus isolates typically consist of multiple strains (1). Seven strains were isolated from a single nucleopolyhedrovirus (SNPV), HaSNPV-AC53 (KJ909666) (2), using a modified tissue culture plaque assay (3, 4). Larvae of *Helicoverpa armigera* were infected with HaSNPV-AC53. Strains were isolated from hemolymph of infected larvae either by plaque purification in HzAM_1_ cells, one passage through tissue culture, and one passage through larvae (“C-strains”), or by passage through tissue culture cells and then one passage of occlusion bodies produced through larvae, followed by plaque purification, passage in cell culture, and one passage through larvae as above (“T-strains”). Viral DNA was extracted from occlusion bodies using a Bioline Isolate II Genomic DNA kit (Bioline, USA) following published methods (2, 5, 6).

Isolated strains and HaSNPV-AC53 were prepared using a NexTera kit (Illumina, USA) and sequenced using the Illumina NextSeq 500 with 150-bp paired-end reads. Trimming was completed using the FASTX-Toolkit version 0.0.13 (7). An eight-step technique to assemble the genomes without gaps was established using a combination of open-source and commercial software. The strains were initially mapped to the HaSNPV-AC53 reference using the Burrows-Wheeler aligner “mem” algorithm (BWA-mem) version 0.7.12 (8) and converted and sorted into the BAM format using SAMtools version 1.2 (9). A gapped-consensus sequence was produced using SAMtools version 1.2, BEDtools2 (10), BCFtools (as part of SAMtools), Picard Tools version 1.140 (http://broadinstitute.github.io/picard) and the Genome Analysis Toolkit version 3.4-46 (11–13). The mapped reads were filtered using bam2fastx as part of TopHat version 2.1.0 (14) and loaded into KmerGenie version 1.6982 (15) to determine the *k*-mer size of the mapped data and then assembled *de novo* using Tadpole (BBMap 35.59 package) (16). The mapped reads, *de novo*-assembled contigs, and the consensus sequence (with gaps) were merged into a single fasta file and mapped against the HaSNPV-AC53 reference using the Geneious R9 mapper with medium-low sensitivity and 5× iterations (17). The final consensus sequence and annotations were completed using Geneious R9.

The HaSNPV-AC53 sequence produced had 100% sequence identity to the published HaSNPV-AC53 reference sequenced on the Ion Torrent PGM (2). One strain was identical in length to the parent HaSNPV-AC53 sequence (130,442 bp): HaSNPV-AC53-C5 (130,442 bp). Four strains were between 5 bp and 7 bp shorter; HaSNPV-AC53-C6 (130,435 bp), HaSNPV-AC53-C9 (130,437 bp), HaSNPV-AC53-T2 (130,437 bp), and HaSNPV-AC53-T5 (130,439 bp). Two strains, HaSNPV-AC53-C3 (130,443 bp) and HaSNPV-AC53-C1 (130,460 bp) were, respectively, 1 bp and 18 bp longer. All the strains contain the 138 open reading frames (ORFs) and 5 homologous repeat regions found within HaSNPV-AC53 (2). Comparison of strain and parent HaSNPV-AC53 sequences shows differences within HOAR, ORF5, ORF7, ORF61, BRO-A, DNA-polymerase, ORF78, 38.7-K protein, ORF128, and PKIP-1, and in all 5 homologous repeat regions. Nonsynonymous mutations were identified in ORF5 (HaSNPV-AC53-C3), BRO-A (HaSNPV-AC53-T2), and DNA-polymerase (HaSNPV-AC53-T2 and HaSNPV-AC53-C5).

### Nucleotide sequence accession numbers.

The complete sequences of HaSNPV-AC53C1, HaSNPV-AC53C3, HaSNPV-AC53C5, HaSNPV-AC53C6, HaSNPV-AC53C9, HaSNPV-AC53T2, and HaSNPV-AC53T5 were deposited to GenBank under the accession numbers KU738896, KU738897, KU738898, KU738899, KU738900, KU738901, and KU738904, respectively.

## References

[1] Blissard GW, Rohrmann GF 1990 Baculovirus diversity and molecular biology. Annu Rev Entomol 35:127–155. doi:10.1146/annurev.en.35.010190.001015. 2154158

[2] Noune C, Hauxwell C 2015 Complete genome sequences of *Helicoverpa armigera* single nucleopolyhedrovirus strains AC53 and H25EA1 from Australia. Genome Announc 3(5):e01083-15. doi:10.1128/genomeA.01083-15. 26404605 PMC4582581

[3] Brown M, Faulkner P 1978 Plaque assay of nuclear polyhedrosis viruses in cell culture. Appl Environ Microbiol 36:31–35. 16345306 10.1128/aem.36.1.31-35.1978 PMC243029

[4] BD Biosciences Plaque assay. http://www.bdbiosciences.com/br/resources/baculovirus/protocols/plaque_assay.jsp. Accessed 16 March 2016.

[5] Rowley DL, Popham HJ, Harrison RL 2011 Genetic variation and virulence of nucleopolyhedroviruses isolated worldwide from the heliothine pests *Helicoverpa armigera*, *Helicoverpa zea*, and *Heliothis virescens*. J Invertebr Pathol 107:112–126. doi:10.1016/j.jip.2011.03.007. 21439295

[6] Baillie VL, Bouwer G 2011 Development of highly sensitive assays for detection of genetic variation in key *Helicoverpa armigera* nucleopolyhedrovirus genes. J Virol Methods 178:179–185. doi:10.1016/j.jviromet.2011.09.009. 21946286

[7] Gordon A, Hannon G 2010 FASTX-Toolkit: FASTQ/A short-reads preprocessing tools. http://hannonlab.cshl.edu/fastx_toolkit. Accessed 16 March 2016.

[8] Li H 2013 Aligning sequence reads, clone sequences and assembly contigs with BWA-MEM. arXiv:13033997. http://arxiv.org/abs/1303.3997.

[9] Li H, Handsaker B, Wysoker A, Fennell T, Ruan J, Homer N, Marth G, Abecasis G, Durbin R, 1000 Genome Project Data Processing Subgroup 2009 The Sequence Alignment/Map format and SAMtools. BioInformatics 25:2078–2079. doi:10.1093/bioinformatics/btp352. 19505943 PMC2723002

[10] Quinlan AR, Hall IM 2010 BEDTools: a flexible suite of utilities for comparing genomic features. BioInformatics 26:841–842. doi:10.1093/bioinformatics/btq033. 20110278 PMC2832824

[11] DePristo MA, Banks E, Poplin R, Garimella KV, Maguire JR, Hartl C, Philippakis AA, del Angel G, Rivas MA, Hanna M, McKenna A, Fennell TJ, Kernytsky AM, Sivachenko AY, Cibulskis K, Gabriel SB, Altshuler D, Daly MJ 2011 A framework for variation discovery and genotyping using next-generation DNA sequencing data. Nat Genet 43:491–498. doi:10.1038/ng.806. 21478889 PMC3083463

[12] McKenna A, Hanna M, Banks E, Sivachenko A, Cibulskis K, Kernytsky A, Garimella K, Altshuler D, Gabriel S, Daly M, DePristo MA 2010 The genome analysis toolkit: a MapReduce framework for analyzing next-generation DNA sequencing data. Genome Res 20:1297–1303. doi:10.1101/gr.107524.110. 20644199 PMC2928508

[13] Van der Auwera GA, Carneiro MO, Hartl C, Poplin R, del Angel G, Levy-Moonshine A, Jordan T, Shakir K, Roazen D, Thibault J, Banks E, Garimella KV, Altshuler D, Gabriel S, DePristo MA 2013 From FastQ data to high-confidence variant calls: the genome analysis toolkit best practices pipeline. Curr Protoc Bioinformatics 11:11.10.1–11.10.33. doi:10.1002/0471250953.bi1110s43. 25431634 PMC4243306

[14] Kim D, Pertea G, Trapnell C, Pimentel H, Kelley R, Salzberg SL 2013 TopHat2: accurate alignment of transcriptomes in the presence of insertions, deletions and gene fusions. Genome Biol 14:R36. doi:10.1186/gb-2013-14-4-r36. 23618408 PMC4053844

[15] Chikhi R, Medvedev P 2014 Informed and automated *k*-mer size selection for genome assembly. BioInformatics 30:31–37. doi:10.1093/bioinformatics/btt310. 23732276

[16] Bushnell B BBMap short read aligner. https://sourceforge.net/projects/bbmap. Accessed 16 March 2016.

[17] Kearse M, Moir R, Wilson A, Stones-Havas S, Cheung M, Sturrock S, Buxton S, Cooper A, Markowitz S, Duran C, Thierer T, Ashton B, Meintjes P, Drummond A 2012 Geneious Basic: an integrated and extendable desktop software platform for the organization and analysis of sequence data. BioInformatics 28:1647–1649. doi:10.1093/bioinformatics/bts199. 22543367 PMC3371832

